# Supplementary material for: Phenotypic memory in quorum sensing
Source: PLoS Comput Biol. 2024 Jul 8;20(7):e1011696. doi: 10.1371/journal.pcbi.1011696 (PMC11257393; doi:10.1371/journal.pcbi.1011696)
Supplement: S1 Text — contains supporting Figs A-G and Table A with concentration values used in simulations. (DOCX) [file pcbi.1011696.s001.docx]

Supplementary information

Phenotypic memory in quorum sensing

Ghazaleh Ostovar, James Q. Boedicker

The values of OFF and ON steady states were obtained from solving the set of ODEs starting from 1 cell/ml, setting the initial conditions corresponding to variables ${I, R, RA, A, A}_{ex}, C,$ and *G* to zero. Within a range of maximum achievable cell densities denoted by $N_{max}$*,* spanning ${10}^{7}-{10}^{12}$ cells/ml, the system exhibited two distinct steady-state solutions contingent upon value of $N_{max}$. These solutions are denoted as the "ON" and "OFF" steady states. Moreover the $N_{max}$ value at which the QS regulated gene, denoted with G, reaches half of its maximum value (corresponding to N_max_ = 10^11^ cells/mL) is defined as the critical cell density required for activation and is shown in red in **Fig A**. **Table A** outlines the concentrations of LuxI, LuxR, monomer, dimer, internal AHL, external AHL, and the QS target gene in both the QS ON and OFF steady states. These values correspond to final cell densities of ${10}^{7}$ and ${10}^{11}$ cell/ml respectively.

**Fig A: Quorum sensing activity state vs. final cell density.** The initial cell density is set to 1 cell/ml, and cells were allowed to grow to final cell densities within the range of ${10}^{7}-{10}^{12}$ cells/ml. The expression of QS-regulated genes shows a significant increase at the critical cell density of ${N_{C}\sim10}^{9.8}$ cells/ml.

**Table A.**

**QS biomolecule concentrations in ON and OFF states.**


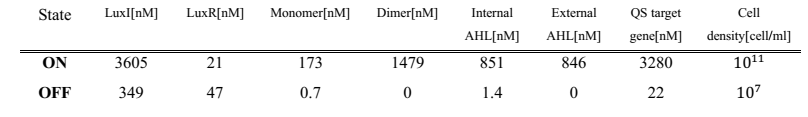


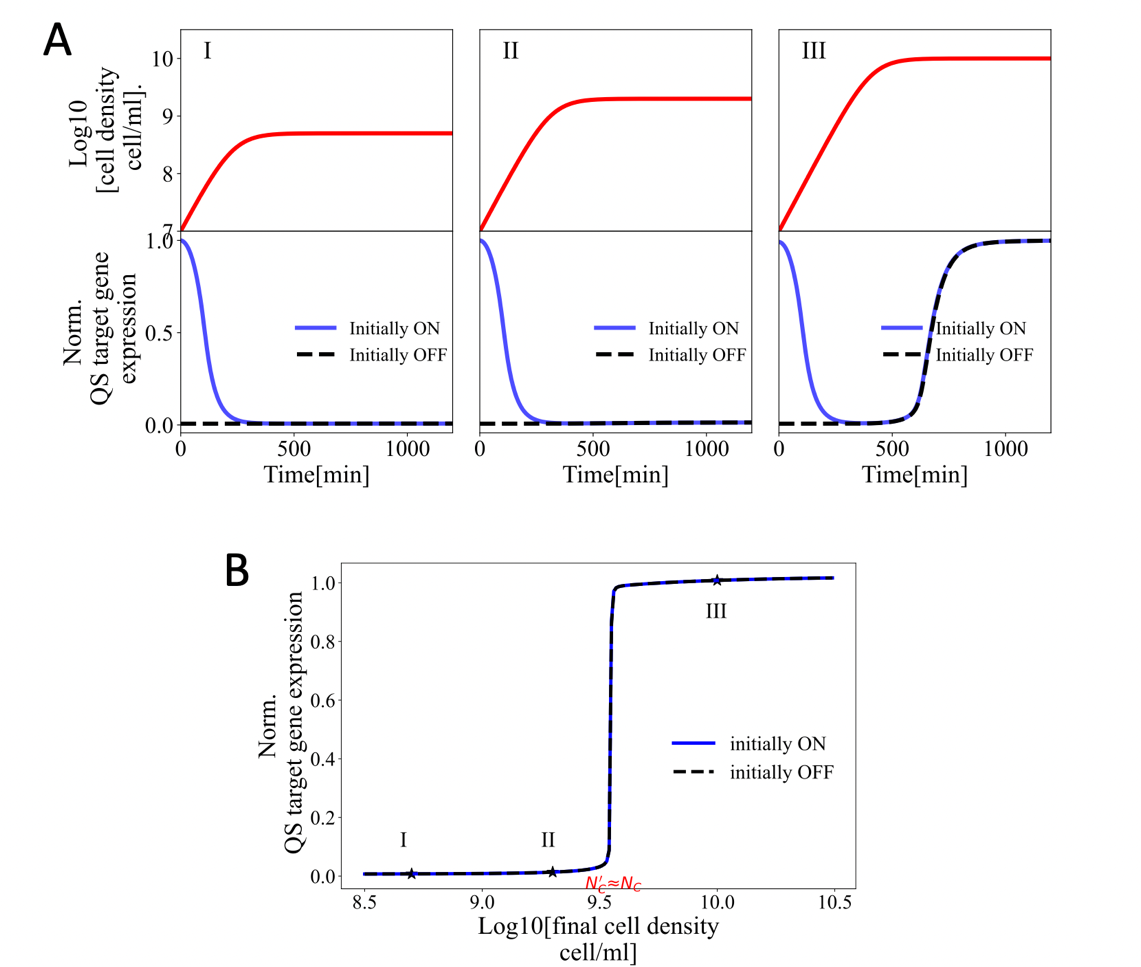


**Fig B. Over-dilution diminishes the memory effect in quorum sensing.**

**A**, top) Populations of cells were grown from a density of ${10}^{7}$ cells/ml to three final cell densities: $I:N_{max}={10}^{8.7}<N_{c}$, $II:N_{max}={10}^{9.3}\approx N_{c}$, and $III:N_{max}={10}^{10}>N_{C}$ cells/ml. **A**, bottom) Normalized QS target gene expression levels for cells initially in the QS ON and QS OFF states, corresponding to each final cell density. **B**) Normalized expression levels of the QS-regulated gene for cells initially in the QS ON and QS OFF states, at the final cell densities in the range of ${10}^{8.5}-{10}^{10.5}$ cells/ml. The low initial cell density leads to an approximate equality in the critical cell density between initially ON and initially OFF cells (Nc' ≈ Nc), diminishing the width of memory zone to zero.


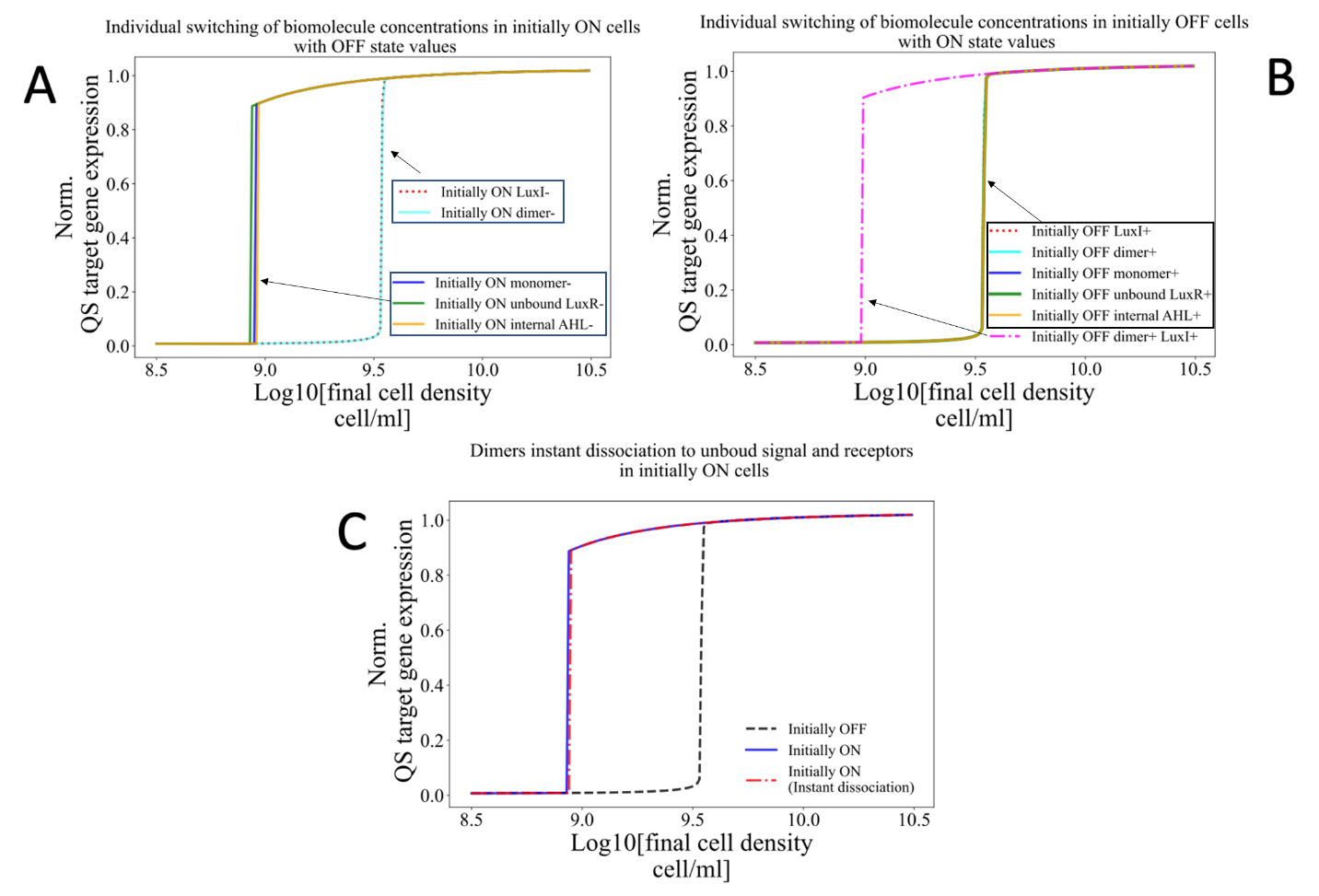


**Fig C. Carry-over effects of individual QS biomolecules on memory zone emergence.** The plot illustrates the final expression level of a QS-regulated gene for cells initially in the QS ON and QS OFF states. Initial cell density of ${10}^{8.5}$ cells/ml, final cell densities vary within the range of ${10}^{8.5}-{10}^{10.5}$cells/ml. **A)** For initially ON cells, the value of each component was switched to OFF values, denoted with a '-' sign in the legend. **B)** For initially OFF cells, the value of each individual component was switched to ON values, denoted with a '+' sign in the legend. **C)** The dashed line corresponds to the scenario where both monomer and dimer concentrations were switched to zero, with their values transferred to unbound receptors and internal AHL molecules (instant dissociation upon dilution). Remarkably, instant dissociation had minimal impact on the activation curve of initially ON cells.

­
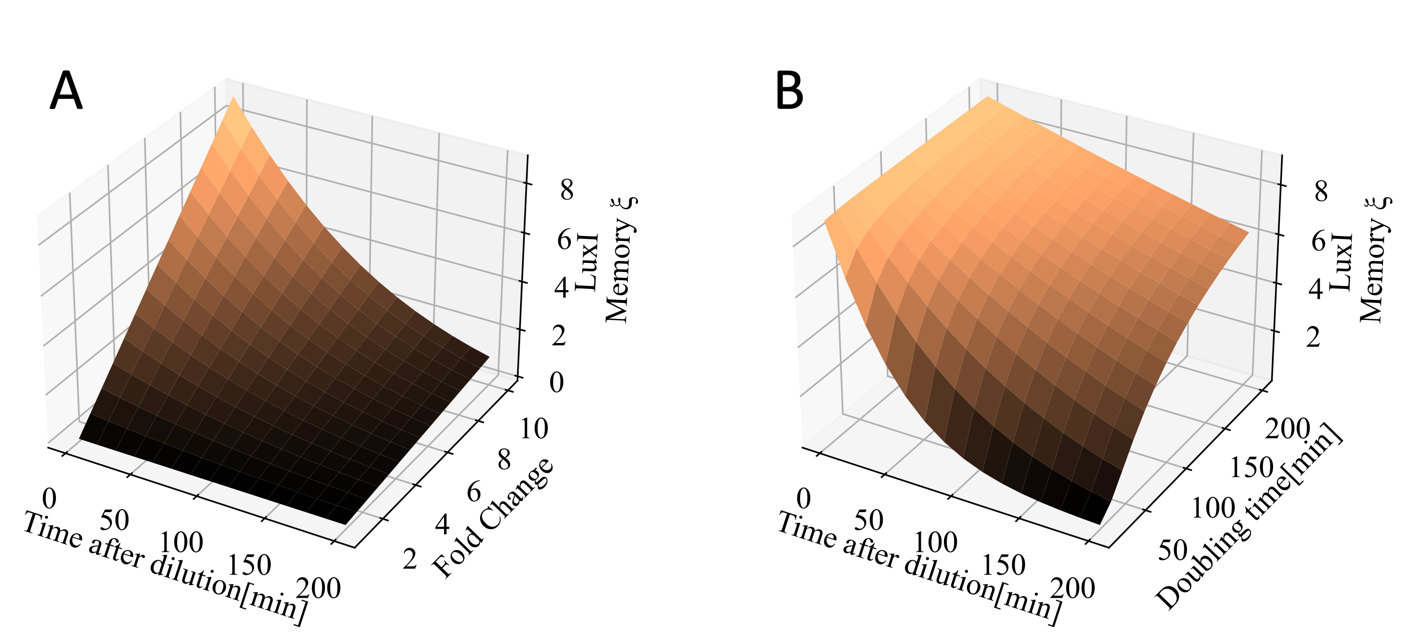


**Fig D. LuxI memory term ξ vs. time after dilution, Fold Change, and doubling time.** **A)** ξ vs. time after dilution, with a fixed doubling time of 40min and varying Fold Change. **B)** ξ vs. time after dilution, with a fixed Fold Change of 10 and varying doubling time.

**
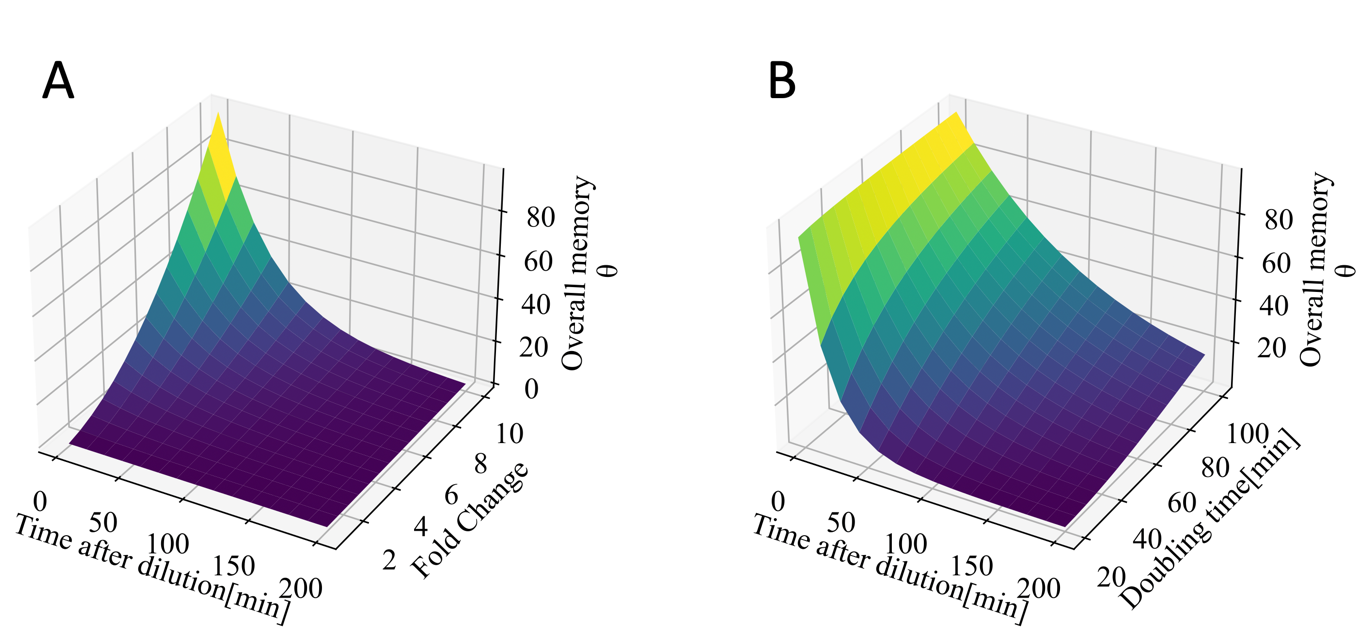
**

**Fig E. Overall memory term Θ in the absence of rapid degradation of LuxR vs. time after dilution, Fold Change, and doubling time.**

**A)** Θ vs. time after dilution and Fold Change. Doubling time is fixed at 40min. **B)** Θ vs. time after dilution and doubling time with a fixed Fold Change of 10 and varying doubling time.

**
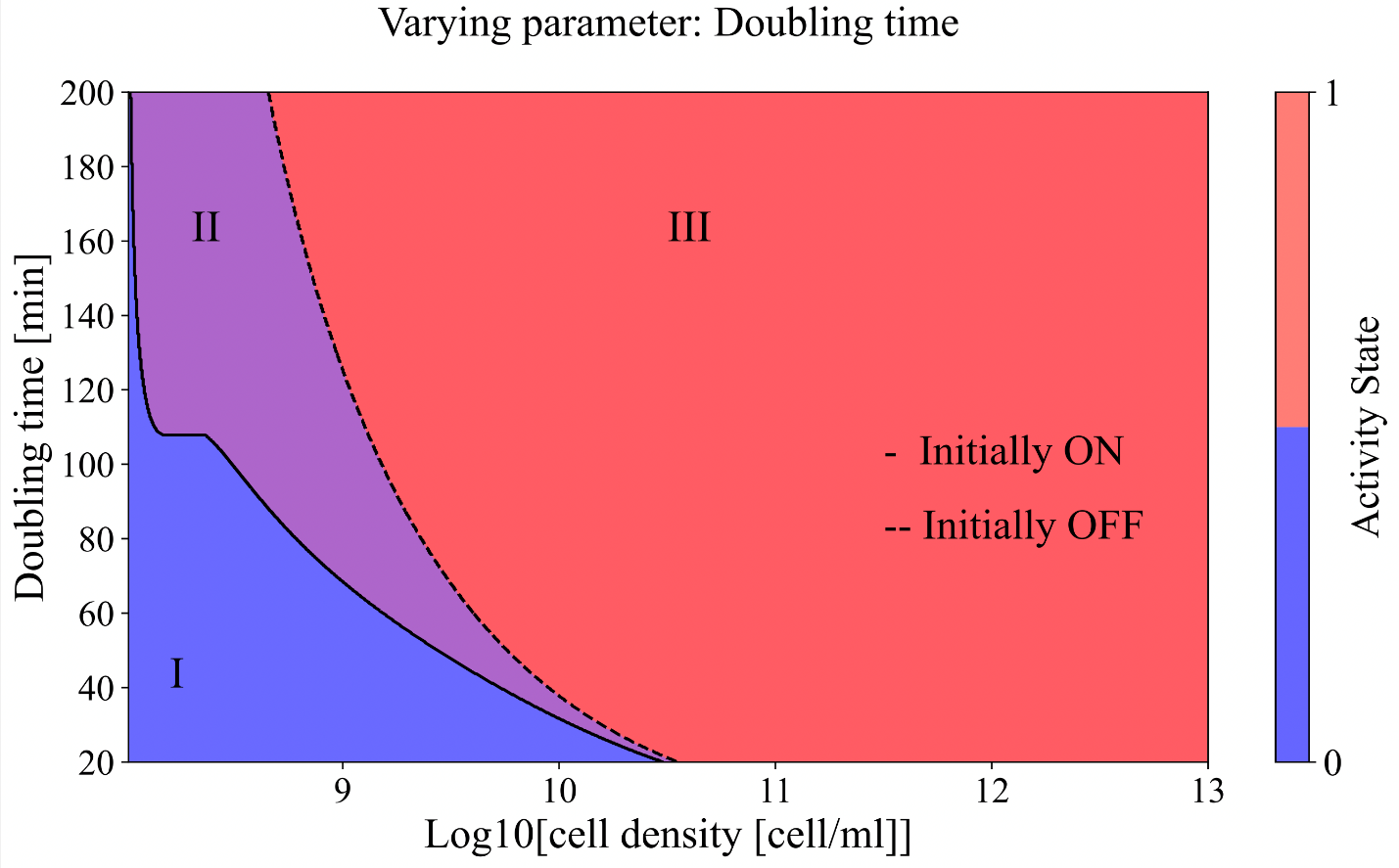
**

**Fig F. The impact of doubling time on memory zone width**. This figure shows the activity states of initially ON and OFF cells within a cell density range from ${10}^{8}$ cells/ml to ${10}^{13}$ cells/ml, all starting from an initial cell density of ${10}^{8}$ cells/ml. Doubling time varies between 20 and 200min. Fold change (*FC*) is set to 10, and the value of $I_{OFF}$ and $\gamma$ were adjusted for each varying doubling time. . In region I (blue) cells are in the OFF state, in region III (pink) cells are in the ON state, and in region II (purple) the activity state depends on the initial activity state. Solid and dashed lines indicate the boundary between regions I and III for cells in initially OFF or ON states. For these calculations $FC=10$, $b=0.04min^{-1}$, $\beta=17 {min}^{-1}$, $A_{threshold}=20nM$, $\gamma_{R}=0.018min^{-1}$, and $v_{cell}={10}^{-12}ml^{-1}$.


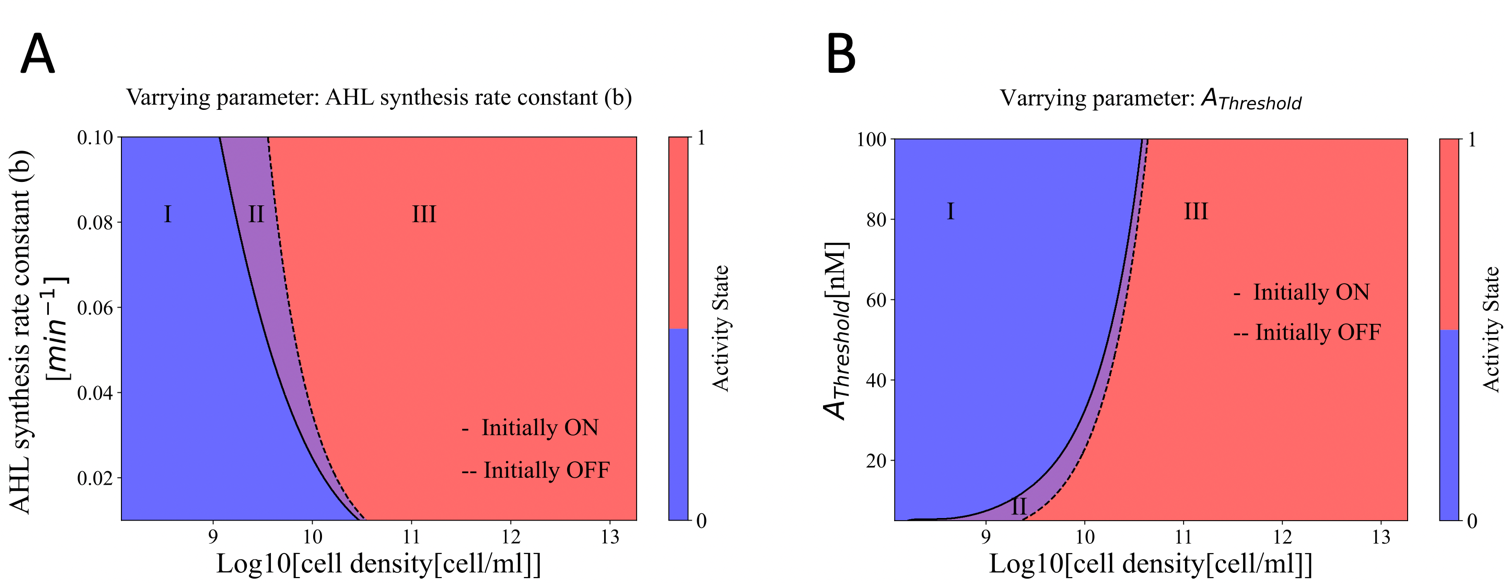


**Fig G. The Effect of the autoinducer synthesis rate (b) constant, and the threshold required for activation (**$\boldsymbol{A}_{\boldsymbol{Threshold}}$**) on memory zone width.**

The figure illustrates the activity states of initially ON and OFF cells. The initial cell density is set to ${10}^{8}$ cells/ml, and with a fixed doubling time of 40 min, cells are allowed to grow for 700min. Fold change (*FC*) is set to 10. . In region I (blue) cells are in the OFF state, in region III (pink) cells are in the ON state, and in region II (purple) the activity state depends on the initial activity state. Solid and dashed lines indicate the boundary between regions I and III for cells in initially OFF or ON states. **A)** $A_{Threshold}$ is maintained at 20nM with a varying autoinducer synthesis rate constant (*b*). **B)** Autoinducer synthesis rate constant (*b*) is set at 0.04$\mathrm{mi}n^{-1}$with varying $A_{Threshold}$. For these calculations $\gamma=017min^{-1}, FC=10$, $I_{OFF}=1000nM$, $\gamma_{R}=0.018min^{-1}$, and $v_{cell}={10}^{-12} ml^{-1}$.
