# Supplementary material for: Phenotypic memory in quorum sensing
Source: PLoS Comput Biol. 2024 Jul 8;20(7):e1011696. doi: 10.1371/journal.pcbi.1011696 (PMC11257393; doi:10.1371/journal.pcbi.1011696)
Supplement: Attachment — Submitted filename: Response to reviewers.docx [file pcbi.1011696.s003.docx]

We sincerely appreciate the detailed and thoughtful comments provided by the reviewers on our manuscript titled "Phenotypic Memory in Quorum Sensing." Your feedback has led to significant improvements, particularly within the Discussion section. Following your suggestions, we have included revisions to clarify our methodologies and strengthen the presentation of our findings. We believe these changes have significantly improved the manuscript and addressed the key concerns raised during the review process. Text that has been added to the revised manuscript are emphasized using red text.

**Reviewer #1: The authors use mathematical modeling to examine the conditions favoring phenotypic memory in QS. Using numerical sims and analytical approximations, the authors show that classical QS architectures (positive feedback control of core QS genes) can produce transient memory effects, in particular a heightened initial sensitivity to re-activation following recent de-activation (dilution). The authors further identify the most important cellular currencies and parameters governing this memory phenomenon.**

**The paper makes a nice logical contribution by showing in a parameterized model that the carry-over of intracellular QS components following dilution can prime the reactivation of QS following subsequent growth. While on some level this ‘has to be true’, the numerical work helps to put some bounds on the importance of this phenomenon, although it has to be said my reading of this is ‘not very important’. Perhaps the authors can push back on this - my reading is that the effects are fairly small, and this is in the favorable case of zero delays (instantaneous dilution, and then immediate entry into favorable growth conditions). It would be good to underline how delays (slower dilution, longer pauses before growth) will rapidly remove all memory effects.**

Thank you for your valuable insights on our manuscript. We appreciate your point regarding the perceived minimal impact of these effects. However, in competitive microbial settings, even subtle shifts in QS activation thresholds can have significant implications for microbial fitness and survival, especially where rapid adaptation is essential. For example, bacteria detaching from a high cell density, QS "ON" environment, and infect smaller, confined spaces, this is akin to high-density conditions. This dispersal, often seen in biofilms, enables bacteria to colonize new surfaces rapidly. Cells from these environments can accelerate new biofilm formation in new settings. Bacteria such as Pseudomonas aeruginosa, which is known for its role in chronic infections, utilize QS to regulate virulence and biofilm formation and can adapt quickly to new environments following dispersion. This adaptability is crucial where rapid colonization is necessary, such as in human lungs during infections. Furthermore, these carry-over effects in QS reactivation could potentially influence the timing and strategy of therapeutic interventions, particularly in QS inhibition strategies.

Additionally, while our model used conservative parameter estimates like a low fold change and rapid growth rate to minimize observed carry-over effects, organisms like *Pseudomonas aeruginosa* can experience fold changes in virulence factors upon QS activation that dramatically exceed our model parameters, potentially amplifying these effects. Slow growth conditions, typical in natural environments like the squid light organ where doubling times can extend beyond a few hours, can further intensify the memory effects associated with carry-over effects.

Regarding entry to favorable growth conditions upon dilution, we agree with the reviewer that delays in bacterial growth can significantly attenuate memory effects. From a molecular standpoint, during growth delays, despite the negligible dilution effect due to cell division, receptor dimers tend to dissociate, and unbound receptors degrade. Consequently, by the time favorable growth conditions resume, a substantial portion of receptor content may have already degraded, nullifying the memory effect. This phenomenon is graphically represented in Supplementary Figure SI 3 A, where we illustrate the minimal memory effect when the initial dimer concentration in ON cells is set to OFF values, emphasizing how both LuxI and receptor dimers critically contribute to the QS memory effect.

In the revised manuscript, we further elaborated on how the degradation dynamics of QS components influence phenotypic memory under varying temporal delays in bacterial regrowth:

Line 617: “In natural quorum sensing (QS) systems, fluctuations in cell density, signal concentration, or both, frequently occur. The nonlinear degradation of LuxR in the absence of signals prevents strong memory effects when there is a delay in growth after perturbation. Although LuxI content might not degrade significantly, as dilution by cell division does not occur, delays in growth still lead to degradation processes where receptor dimers dissociate, and unbound receptors degrade. Consequently, by the time favorable growth conditions resume, much of the receptor content may have already degraded, particularly if delays are prolonged, thereby nullifying the memory effect.”

**2. Concerning the importance and impact of this work – I suspect that establishing the importance (or not) of QS memory will require experimental investigation. Adding some words on experimental tests that follow from this work would be helpful.**

Thank you for your insightful comments, which underscore the necessity of experimental validation to complement our theoretical and simulation-based study on quorum sensing (QS) memory. We acknowledge the significance of empirical evidence to substantiate our model predictions and appreciate your suggestions for outlining potential experimental validations.

Line 701: “Building upon previous experiments demonstrating the presence of phenotypic memory in bacteria [17], we propose to extend these studies to measure the effects of molecular carry-over in cells previously activated ('ON' state) compared to naive ('OFF') cells. Prior work has shown that when a high concentration of signal is added to cells previously in a quorum sensing active state, the carry-over effects led to a faster reactivation of quorum sensing [17]. Future work should further probe quorum sensing memory, testing if carry-over of synthase proteins also changes reactivation dynamics, as predicted in this study. Experimentally probing the limits of QS memory would help determine the specific contexts in which this memory has an impact. To specifically investigate the effects of each biomolecule, the synthase and receptor proteins, genetic circuits could be designed to modulate the Fold Change and degradation rates of each protein independently. To closely mimic the environmental fluctuations that bacterial communities naturally encounter, we recommend exposing bacterial populations to periodically changing signal concentrations and cell densities using microfluidic chambers. Employing microfluidic platforms will enable precise control over experimental conditions [11, 56], providing insights into how QS systems adapt over time to dynamic signal environments. The role of cell division and protein degradation in QS memory should also be explored, as these are key factors that set the duration of phenotypic memory. Our findings demonstrated that systems with a high Fold Change, low activation threshold, and high basal levels of signal synthesis exhibit the strongest memory effects, and these results could be experimentally validated. Such studies will not only corroborate our theoretical findings but also expand our understanding of microbial communication in fluctuating environments, potentially leading to novel strategies for managing microbial populations.”

**3. Methods / Model structure: please state what bug and what environment you are modeling. Table 1 has specific parameters which imply a specific experimental setting defined at a minimum by a species and a medium.**

Thank you for your question regarding the specifics of our model. We have revised the text to give more details regarding our system and experimental context. The dissociation rates (k_-1, k_-2) and constants associated with monomer and dimer formation (kd1, kd2) were directly sourced from the literature, as cited. The basal and activated expression rates of LuxI and LuxR (k5, k6, k3, k4), along with the basal expression rate of the QS target gene (k10) were set considering literature values, adjusted to reflect measured signal concentrations and cell density activation thresholds. The values of these parameters used the model achieve quorum sensing activation around 10^9^ cells/mL. The following was revised in the main text:

Line 123: “Although the model should apply to many quorum sensing systems, it is inspired by the LuxR_LuxI circuit of *Vibrio fischeri*, a well-studied marine bacterium known for its bioluminescent interaction with the Hawaiian bobtail squid. This choice is based on the extensive availability of quantitative data regarding the QS system of *Vibrio fischeri*.”

Line 155: “**Table 1:** Model parameters. *Estimated based on [32], LuxI and LuxR are assumed to have approximately the same basal expression rates and QS activation at a cell density near 10^9^ cell/mL. ** Rate constants are estimated to achieve a Fold Change of 10 for LuxI ***Estimated to obtain half maximum activity at around 25-50 nM AHL concentration [33].”

**4. Thinking of signal degradation – many bugs have active QS-controled processes of signal degradation (lactonases, acylases etc) – this might be a design feature to re move memory effects? Possibly something to consider in the discussion?**

Thank you for your attentive comment on deliberate signal degradation in bugs. One notable impact of this degradation is the delayed activation of quorum sensing (QS) pathways, which is expected to diminish memory effects. We have elaborated on this in our discussion as follows:

Line 624: “In quorum sensing (QS) systems, cell density perturbations can occur while external signal concentrations remain unchanged. Enzymes like lactonases and acylases degrade signaling molecules [53], leading to a delayed activation of QS pathways and a reduction in memory effects. These enzymes act as a natural "reset" mechanism, modulating autoinducer levels to prevent the reactivation of QS pathways from residual high-density signals. This adjustment avoids unnecessary energy or virulence factor production and aligns QS responses more dynamically with the current environmental and cell density conditions.”

**5. Line 132: I find the choice of a continuous growth assumption confusing, when elsewhere in the same model you have a saturating logistic growth function. Why not couple growth dilution to your explicit growth equation?**

Thank you for your insightful observations regarding the growth assumptions employed in our model. The sentence on the original line 132 was potentially confusing. We have revised that section of the text to clarify the assumptions related to protein degradation:

Line 129: “The protein concentration per cell is reduced due to both cell division and protein decay. The rate of protein degradation was set to reflect that cell division sets the rate that proteins are diluted during cell growth. To simplify the model, this rate of protein degradation does not change as cell growth slows near the saturating cell density. Separate degradation and production rates could have been implemented, dependent on the growth phase of the culture, however QS activation is expected to occur well before reaching density-dependent growth limitations. This simplification prevents a ramp up in protein levels during stationary phase. While dilution resulting from cell division is often the primary mechanism of protein degradation, it should be noted that LuxR is inherently unstable in its non-dimeric state, particularly when AHL concentrations are low [25], and thus is prone to rapid degradation.”

**Reviewer #2: The paper "Phenotypic memory in quorum sensing" explores the concept of memory in bacterial quorum sensing (QS) systems. It discusses how bacteria retain information about past environmental conditions through a mechanism known as phenotypic memory, which impacts their future behavior in response to stimuli like cell density and extracellular signal concentrations. Through mathematical modeling and simulations, the authors investigate how various cellular parameters affect the strength and duration of this memory in QS systems. I recommend for its publication if the following concerns could be addressed adequately.

1. The paper provides insights into the transient effects of QS memory but lacks a deep mechanistic explanation of how specific cellular components contribute to this memory.
Suggestion: Expand the discussion on the role of different QS components in memory formation, particularly how proteins like LuxR and LuxI contribute to memory persistence. Include a detailed analysis or additional simulations that isolate the effects of individual components on memory strength and duration.**

Thank you for your constructive feedback. We note that SI Figure 3 examines the contribution of different cellular components to QS memory. To bring attention to this point, we have highlighted the findings of this figure and expanded our discussion about this topic:

Line 300: “we switched the value of each component in the initial conditions of the initially ON cells individually to OFF values and plotted the corresponding activation curve as shown in **Fig. S3A.** This figure analyzed the isolated role of different molecular components on QS memory. Switching either dimers or LuxI to OFF values, leads to significant reduction in the memory zone, resulting in an activation curve that overlaps the initially OFF cells.”

Expanded Discussion starting at Line 576: “Activation at lower critical cell densities is largely due to the carry-over of synthase proteins and receptors, which are predominantly in dimeric form in initially ON cells. An excess of synthases in these cells leads to an earlier accumulation of signals above baseline levels compared to initially OFF cells. This increased signal concentration boosts the rate of receptor binding reactions, potentially facilitating the formation of new dimers necessary for reactivation. Moreover, an initial surplus of receptors in ON cells enhances their sensitivity to current signal concentrations by accelerating receptor binding reactions, thus potentially enabling activation at lower cell densities compared to OFF cells. Furthermore, simulation results indicate that both LuxR, mainly in its stable dimeric form, and LuxI play critical roles in sustaining the memory effect by priming the quorum sensing (QS) switch. Substituting either the dimers or LuxI molecules with those from OFF cells results in a significant reduction in the memory effect. Additionally, replacing both LuxI and dimers with ON values in initially OFF cells led to earlier QS activation, emphasizing the role of these components in hastening the QS response. Interestingly, the presence of excess LuxR was found to be a crucial factor for earlier activation, irrespective of its configuration. Substituting dimeric LuxR with unbound forms did not alter the outcomes, suggesting that the availability of LuxR, rather than its dimeric configuration, plays a pivotal role in the activation process. We have shown that this molecular carry-over affects the reactivation specifically near the critical cell density required for activation, where the time required for activation is smaller.”

**2. The study is theoretical and heavily relies on simulations. Reference to experimental results and proposals of future experimental validations would be helpful.**

Thank you for your valuable feedback. Both reviewers have highlighted a similar concern regarding the need for empirical evidence to complement and validate the theoretical models and simulations presented in this study. We appreciate the emphasis on the importance of experimental work in substantiating the theoretical insights into quorum sensing (QS) memory and have added suggestions for future experiments to the Discussion:

Line 701: “Building upon previous experiments demonstrating the presence of phenotypic memory in bacteria [17], we propose to extend these studies to measure the effects of molecular carry-over in cells previously activated ('ON' state) compared to naive ('OFF') cells. Prior work has shown that when a high concentration of signal is added to cells previously in a quorum sensing active state, the carry-over effects led to a faster reactivation of quorum sensing [17]. Future work should further probe quorum sensing memory, testing if carry-over of synthase proteins also changes reactivation dynamics, as predicted in this study. Experimentally probing the limits of QS memory would help determine the specific contexts in which this memory has an impact. To specifically investigate the effects of each biomolecule, the synthase and receptor proteins, genetic circuits could be designed to modulate the Fold Change and degradation rates of each protein independently. To closely mimic the environmental fluctuations that bacterial communities naturally encounter, we recommend exposing bacterial populations to periodically changing signal concentrations and cell densities using microfluidic chambers. Employing microfluidic platforms will enable precise control over experimental conditions [11, 56], providing insights into how QS systems adapt over time to dynamic signal environments. The role of cell division and protein degradation in QS memory should also be explored, as these are key factors that set the duration of phenotypic memory. Our findings demonstrated that systems with a high Fold Change, low activation threshold, and high basal levels of signal synthesis exhibit the strongest memory effects, and these results could be experimentally validated. Such studies will not only corroborate our theoretical findings but also expand our understanding of microbial communication in fluctuating environments, potentially leading to novel strategies for managing microbial populations.”

**3. The conclusion discusses the implications of phenotypic memory in QS but does not fully integrate these findings into the broader context of bacterial behavior and evolution. It would be helpful if the authors could elaborate on how QS memory might influence bacterial population dynamics, virulence, and adaptation in fluctuating environments. Discuss potential evolutionary advantages or disadvantages conferred by phenotypic memory in QS systems.**

Thank you for your constructive feedback. We appreciate your suggestion to broaden the discussion of phenotypic memory in quorum sensing (QS) within the wider context of bacterial behavior and evolution. We have expanded the Discussion section accordingly to address these aspects more comprehensively:

Line 605: “Although phenotypic memory has been shown to provide advantages in fluctuating environments by optimizing long-term fitness in metabolic regulatory systems [11, 45, 46], its implications within QS systems remain unclear. It has been shown that in fluctuating environments, the optimal strategy for achieving fitness may vary depending on the temporal order and speed of environmental changes. For example, it has been shown that slower or diversified responses is preferred in rapidly changing environments, potentially surpassing strong memory effect [46-50]. The potential disadvantages of QS memory might arise when environmental correlations that historically informed bacterial responses become decoupled due to these rapid environmental changes. In such scenarios, memory-based responses might no longer confer the appropriate adaptive advantages, leading to reduced fitness and possibly increased extinction risks [52]. Additionally, the ability to adapt to changing environments often comes with a fitness cost for growth in stable environments [47].”

Line 632: “Phenotypic memory in QS is presumably advantageous in periodically fluctuating environment. The disruption of quorum sensing activity through the removal of external autoinducers and reduction of cell density imposes a metabolic cost for re-synthesis of autoinducers [45] until reactivation occurs. In such cases, carry-over effects can be advantageous, enabling cells to initiate a subsequent response at a lower metabolic cost and maximize fitness in the new environment. This memory effect is most prominent when the initial cell density is close to the critical cell density required for activation, ensuring that phenotypic memory is noticeable only when the reduction in cell density is relatively small. Moreover, research has demonstrated that in environments with extreme and random fluctuations, adopting a mixed strategy is often the most effective for survival [15]. Phenotypic memory, in this context, resembles such a mixed strategy, enhancing survivability in unpredictably changing environments. Ecologically, even transient phenotypic memory can influence interspecies relationships, potentially altering the pattern of associations between species. Additionally, it suggests that the phenotypic memory of species is likely to co-evolve [15]. In environments subject to frequent changes, bacteria equipped with advanced QS memory capabilities can gain a distinct competitive advantage. This allows them to activate survival strategies and dominate ecological niches. Such advantages are especially critical during abrupt environmental changes that demand rapid responses for survival. Consequently, QS memory not only boosts the fitness of individual bacteria but also affects community-level interactions and stability, potentially leading to the development of dominant bacterial strains finely adapted to their fluctuating environments.

Regardless of whether phenotypic memory in QS serves an evolutionary adaptive advantage, it is crucial to study this phenomenon due to various implications in industry and medicine, including QS inhibitors. An example of this is bacteria detaching from a high cell density, QS "ON" environment and infecting small, confined spaces resembling high cell density conditions. This phenomenon is particularly observed in biofilms, where bacteria can disperse from a mature biofilm to colonize new surfaces. The dispersed cells can exhibit behaviors influenced by their previous high cell density environments, potentially accelerating the establishment of new biofilms in confined areas. Phenotypic memory in QS affects population dynamics by enabling faster responses to environmental changes, enhancing competitive advantages in resource utilization and colonization. Moreover, QS memory potentially equips bacteria to adapt more rapidly to environmental fluctuations, which can be crucial for survival in dynamic conditions such as nutrient availability and host immune responses. In pathogenic bacteria, QS memory can modulate the timing and intensity of virulence factor production, potentially affecting their ability to evade or suppress host defenses. Bacteria like *Pseudomonas aeruginosa*, known for its role in chronic infections, often utilize quorum sensing to regulate virulence and biofilm formation, adapting quickly to new environments after dispersing from their original colony. This adaptability is crucial in environments where rapid colonization is necessary for survival, such as in the human lungs during infection. Furthermore, the carry-over effects in QS reactivation can potentially influence the timing and strategy of therapeutic interventions, particularly in QS inhibition approaches. Phenotypic memory could be crucial in strategies for managing infections or treatments, especially considering the frequency and predictability of the environmental pressures involved.”
